# Long non-coding RNAs involved in autophagy regulation

**DOI:** 10.1038/cddis.2017.464

**Published:** 2017-10-05

**Authors:** Lixian Yang, Hanying Wang, Qi Shen, Lifeng Feng, Hongchuan Jin

**Affiliations:** 1 Laboratory of Cancer Biology, Key Lab of Biotherapy in Zhejiang, Sir Runrun Shaw Hospital, Medical School of Zhejiang University, Hangzhou, China; 2 Department of Radiation Oncology, Anhui Provincial Hospital, Anhui Medical University, Anhui, China

## Abstract

Autophagy degrades non-functioning or damaged proteins and organelles to maintain cellular homeostasis in a physiological or pathological context. Autophagy can be protective or detrimental, depending on its activation status and other conditions. Therefore, autophagy has a crucial role in a myriad of pathophysiological processes. From the perspective of autophagy-related (*ATG*) genes, the molecular dissection of autophagy process and the regulation of its level have been largely unraveled. However, the discovery of long non-coding RNAs (lncRNAs) provides a new paradigm of gene regulation in almost all important biological processes, including autophagy. In this review, we highlight recent advances in autophagy-associated lncRNAs and their specific autophagic targets, as well as their relevance to human diseases such as cancer, cardiovascular disease, diabetes and cerebral ischemic stroke.

## Facts

Autophagy degrades non-functioning or damaged components of cells to maintain cellular homeostasis and thus has a very significant role in cell development, differentiation and death.
LncRNAs have emerged as non-canonical regulators that take part in a collection of pathophysiological processes, including autophagy, by directly binding to RNA, DNA or protein.
LncRNAs generally modulate autophagy via regulating the expression of *ATG* genes. They often function as competing endogenous RNAs (ceRNAs) to modulate autophagy-related microRNAs (miRNAs).
Autophagy may, in turn, regulate lncRNA expression, although only one such lncRNA has been found so far.

## Open Questions

Can autophagy-related lncRNAs directly regulate *ATG* genes apart from indirect modulation via miRNAs or epigenetic modification enzymes, and if so, how?
Among the several *ATG* genes that are simultaneously regulated by one autophagy-related lncRNA, is there a chief gene that determines the occurrence or repression of autophagy?
How does autophagy regulate the levels of lncRNAs? Does it degrade them directly in a selective manner or regulate them indirectly?

LncRNAs are non-coding RNAs longer than 200 nucleotides.^1, 2^ They have recently been found to have a crucial role in various fundamental physiopathologic processes, such as carcinogenesis, as well as autoimmune, cardiovascular and neurological diseases.^3, 4, 5^ LncRNAs can be classified according to their gene locations or functions. On one hand, lncRNAs can be named after their gene loci relative to adjacent protein-coding genes, which are antisense lncRNAs, intronic lncRNAs, bidirectional lncRNAs and intergenic lncRNAs (also named lincRNAs).^6, 7, 8, 9^ On another, lncRNAs can act as decoys, scaffolds, guides and enhancers to regulate DNA, RNA and proteins, on the basis of their function.^10, 11, 12, 13, 14^ In addition, lncRNAs have recently been documented to serve as competing endogenous RNAs (ceRNAs), which can sequester microRNAs (miRNAs) from targeted mRNAs sharing the same miRNA response elements (MREs), thereby regulating the expression of the targeted mRNAs.^15^ Owing to the increasing number of functionally characterized lncRNAs, the classification is constantly being updated. In this review, we will summarize the functions of lncRNAs involved in autophagy, a self-digestive process that helps to maintain cellular homeostasis.^16, 17^

Macroautophagy (hereafter referred to as autophagy) is a process that delivers cytoplasm components, enclosed in double-membrane vesicles, to lysosomes for degradation.^16^ By doing so, autophagy has a critical role in maintaining cellular homeostasis in response to various environmental stresses, such as nutrient deprivation and hypoxia, as well as chemical and physical damage.^16, 17^ Therefore, autophagy is crucial in various pathological and physiological processes such as immunity, cancer, cardiovascular diseases and neurodegenerative diseases.^18, 19, 20^

**Figure 1:**
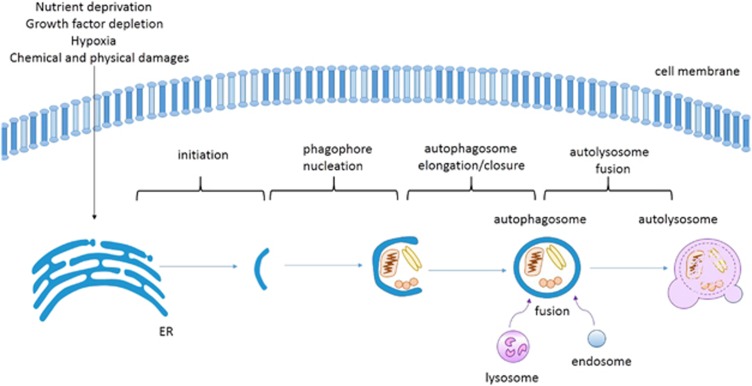
The simple model of autophagy pathway. Diversities of stimuli for autophagy (such as amino-acid starvation, growth factor depletion, hypoxia, chemical and physical damage) initiate formation of the isolation membrane (or ‘phagophore’) from endoplasmic reticulum (ER, the best recognized origin for autophagosome membrane). Subsequently, the isolation membrane encloses cytoplasmic components as it elongates sequentially and its closure lead to the birth of ‘autophagosome’. Then, autophagosome fuses with endosomes and lysosomes to form autolysome in which the enclosed cytoplasmic materials are degraded by lysosomal

To date, at least 37 autophagy-related (*ATG)* genes are known to exist in yeast, and many of their orthologs have been identified in mammals.^21, 22^ We will discuss the crosstalk between autophagy-related lncRNAs and various *ATG* genes based on the four well-defined steps of autophagy: initiation, phagophore nucleation, autophagosome elongation/closure and autolysosome fusion^23^ (Figure 1). Certainly, lncRNAs may target several *ATG* genes at the same time and thus regulate autophagy at different stages. In this case, we will classify them into the category of most relevant stage.

## LncRNAs Involved in Regulating Autophagy via *ATG* Genes

### LncRNAs related to autophagy initiation

Adenine monophosphate-activated protein kinase (AMPK) and mammalian target of rapamycin complex 1 (mTORC1) are two major proteins that sense external stimuli for autophagy initiation.^24, 25, 26^ Upon energy limitation, AMPK is phosphorylated and activated by increased AMP to inhibit mTORC1 and to activate the ATG1/ULK1/2 (yeast/mammal) complex, leading to the initiation of autophagy.^24, 25, 26^ In addition, mTORC1 can be activated by class I phosphatidylinositol 3-kinase (PI3K)/AKT signaling, and thus the PI3K/AKT/mTOR pathway is negatively linked to autophagy initiation.^27, 28^ Once autophagy is initiated, ATG17 forms a complex with ATG29 and ATG31, and then interacts with ATG1 and ATG13 to mediate the assembly of pre-autophagosomal structure (PAS), (the site of autophagosome formation) in yeast.^29, 30^ Similarly, in mammalian cells, ULK1/2 (mammal ortholog of ATG1) forms a stable complex with ATG13, FIP200/RB1CC1 and ATG101 and is subsequently transferred to omegasomes (the cradle for autophagosome biogenesis).^31, 32^

A study reveals that high glucose reduces the level of lncRNA *H19*, which subsequently relieves the transcriptional repression of DIRAS3, and ultimately induces autophagy initiation by repressing PI3K/AKT/mTOR pathway.^33^ As marked activation of autophagy by high glucose has been shown to be detrimental to cardiac function, overexpression of *H19* helps to alleviate this tendency.^33^ In addition, downregulation of *H19* can increase Beclin 1 (mammal orthologous of ATG6) and ATG7 expression, which is a good indicator for further investigation of the association between *H19* and autophagy.^33, 34^ Contrarily, another study has found that exogenous overexpression of *H19* induces autophagic cell death in cerebral ischemia and reperfusion (I/R) injury.^34^ Researchers confirmed that *H19* induces autophagy by inhibiting DUSP5, a mitogen-activated protein kinase phosphatase. DUSP5 is known to repress phosphorylation of ERK1/2, and activation of the latter has been reported to provoke autophagy.^35^ The opposite effects of *H19* on autophagy in diabetic cardiomyopathy and cerebral I/R injury indicate that conditional gene interference with *H19* may be an efficient therapeutic approach to different pathological processes.

A recent study suggests that the lncRNA neighbor of BRCA1 gene 2 (*NBR2*) can bind to AMPK and promote its activation. Intriguingly, the expression of *NBR2* also can be induced by the increasing activation of AMPK under energy stress. Thus, AMPK/*NBR2* forms a feed-forward loop to sustain AMPK activation in response to energy stress, which is a protective factor for normal cells to resist tumors.^36, 37^ Indeed, *NBR2* expression is reduced in human cancers, and loss of its function is correlated with poor prognosis for cancer patients.^36, 38, 39^ This study serves as an interesting example illustrating that lncRNAs can regulate protein activation through direct binding. Future work is needed to demonstrate this functional model of lncRNAs and to address how it takes effect: through changing the conformation of the protein or altering its affinity for other regulatory factors? Another study demonstrates that miR-19a can negatively regulate *NBR2* and AMPK, probably by base pairing between miR-19a and *NBR2* or miR-19a and PRAA1 (the gene encoding AMPK).^40^ The upregulation of miR-19a in acute liver failure (ALF) results in the downregulation of *NBR2* and AMPK expression, which represses autophagy. In light of the protective role of autophagy in the progression of ALF, activation of the miR-19a-*NBR2*/AMPK regulatory axis exacerbates ALF.^40^ This study gave rise to the hypothesis of a ceRNA network in which NBR2 might act as a sponge for miR-19a and prevent it from binding to AMPK mRNA.

As a multitargeted tyrosine kinase inhibitor (TKI), sorafenib has been demonstrated to induce autophagic hepatocellular carcinoma (HCC) cell death, and thus the blockage of autophagy facilitates sorafenib resistance in HCC.^41, 42^ Several miRNAs, including miR-21, miR-216a, miR-217 and miR-494, have been shown to confer sorafenib resistance in HCC by inhibiting autophagy through targeting phosphatase and tensin homolog (PTEN).^42, 43, 44^ For the sake of reversing sorafenib resistance, an artificial long non-coding RNA (AlncRNA), Ad5-AlncRNA, was constructed to target these miRNAs simultaneously.^45^ Overexpression of Ad5-AlncRNA in HCC can sequester these miRNAs from binding to the 3'-UTR of PTEN mRNA. As a result, PTEN is upregulated to repress AKT/mTOR activity and then activates autophagy, sequentially reversing sorafenib resistance.^45^ Thus, artificial lncRNAs targeting several miRNAs of the same mRNA will be a potent therapeutic strategy for diseases.^45, 46^

IFN-*γ* induced by *Mycobacterium bovis* BCG can repress lncRNA *maternally expressed gene 3* (*MEG3*), which decreases p70-S6K (Thr389) phosphorylation subsequently, a downstream factor of mTOR complex, indicating mTOR inactivation. As a result, autophagy is activated and eradication of intracellular *Mycobacterium bovis* BCG is enhanced.^47^ Similar to pathogen infection in macrophages, tumor cells may also trigger autophagy to survive under various stresses through repressing *MEG3*. Indeed, the *MEG3* expression level is significantly reduced in bladder cancer cells, leading to increased autophagy flux and cell proliferation.^48^ In addition, *MEG3* has also been found to promote cisplatin-induced apoptosis by inhibiting autophagy in human glioma cells.^49^ By contrast, another study shows that overexpression of *MEG3* induces autophagy, thereby repressing tumorigenesis and progression of epithelial ovarian carcinoma by regulating ATG3. *MEG3* can bind to ATG3 protein and protect ATG3 mRNA from degradation.^50^ However, more work is needed to elucidate how *MEG3* modulates ATG3 protein and mRNA.

Studies have demonstrated that downregulation of the lncRNA *HOTARIM1* can block autophagy, and thus inhibit all-trans retinoic acid (ATRA)-induced autophagic degradation of PML-RARA in promyelocytic leukemia (PML) cells.^51, 52, 53^ *HOTAIRM1* may provoke autophagy by preventing miR-20a/106b and miR-125b from decreasing ULK1, E2F1 and DRAM2 expression, thus contributing to autophagy-dependent degradation of PML-RARA.^53, 54, 55, 56^ This indicates that overexpression of *HOTAIRM1* might be a potential therapeutic measure for PML.

The lncRNA *PTENP1* shares a similar 3'-UTR with the tumor-suppressor gene *PTEN*, and protects *PTEN* from miRNA-mediated silencing.^57^ Sustained *PTENP1* expression in HCC cells upregulates *PTEN* expression and suppresses the PI3K/AKT signaling cascade, which results in the induction of autophagy, as well as the inhibition of cell proliferation and migration/invasion.^58^ In addition, *PTENP1* can antagonize miR-17 and miR-20a to enhance the expression of ULK1, ATG7 and p62/SQSTM1 (sequestosome 1).^58, 59, 60^ Furthermore, overexpression of *PTENP1* can suppress mTOR phosphorylation and downregulate Bcl-2 expression.^58^ Nevertheless, it remains to be determined whether molecules in this complicated regulatory network are directly regulated by PTENP1 or indirectly altered secondary to some initial factors. Interestingly, researchers have constructed Sleeping Beauty (SB)-based hybrid baculovirus (BV) vectors for sustained *PTENP1* expression.^58^ This system could be a promising instrument for endogenous overexpression of lncRNAs in specific tissues for therapeutic purposes.

The lncRNA *regulator of insulin sensitivity and autophagy* (*Risa)* can affect insulin sensitivity by altering autophagic activity. Indeed, knockdown of *Risa* increases the phosphorylation of ULK1 (Ser757), which contributes to autophagy activation and attenuates insulin resistance.^61^ Unfortunately, there are still divergent schools of thought regarding what role autophagy has in insulin resistance or diabetes.^62, 63^ Thus, whether knockdown of *Risa* can alleviate insulin resistance by promoting autophagy in patients with diabetes requires further exploration.

The lncRNA *AK156230* has been found to repress replicative senescence (RS) in mouse embryonic fibroblasts (MEFs), and one of the downstream pathways involved is autophagy.^64^ Pathway analysis shows that the mTOR signaling pathway is associated with *AK156230* knockdown, and transcriptional levels of *ATG* genes including *ULK2*, *ATG7* and *ATG16L* apparently decrease accordingly.^64^ Vague though the facts are, *AK156230* seems to participate in autophagy induction, as its downregulation results in a deficiency of autophagy, which may accelerate aging.^65^

Another lncRNA, *metastasis-associated lung adenocarcinoma transcript 1* (*MALAT1*), has attracted a great deal of interest for its elusive role in autophagy regulation.^66, 67, 68, 69, 70, 71^ *MALAT1* is upregulated and acts as a sponge for miR-26b to upregulate its target *ULK2* in cerebral I/R injury.^71^ In light of the protective effect of autophagy against I/R in brain microvascular endothelial cells, the enhanced activity of the *MALAT1*/miR-26b/ULK2 regulatory axis appears to be a self-defense mechanism in response to ischemic stroke, the pathological basis of which is I/R injury.^71^ Another study demonstrates that *MALAT1* silencing notably elevates p62 and decreases LAMP2 expression, which is essential for the fusion of autophagosomes and lysosomes.^66^ Moreover, *MALAT1* can act as a 'sponge' for miR-216b to induce autophagy, probably through derepressing Beclin 1 expression, which, in consequence, ameliorates the multidrug resistance of HCC cells.^67, 68^ Interestingly, a newly published article reports that *MALAT1* knockdown can indeed reduce the expression of Beclin 1 in multiple myeloma tissues, although the details of the mechanism are not known.^69^ In contrast to the studies mentioned above, *MALAT1* inhibition has been found to induce autophagy in diffuse large B-cell lymphoma (DLBCL) cells and improve its response to adriamycin treatment.^70^

**Figure 2:**
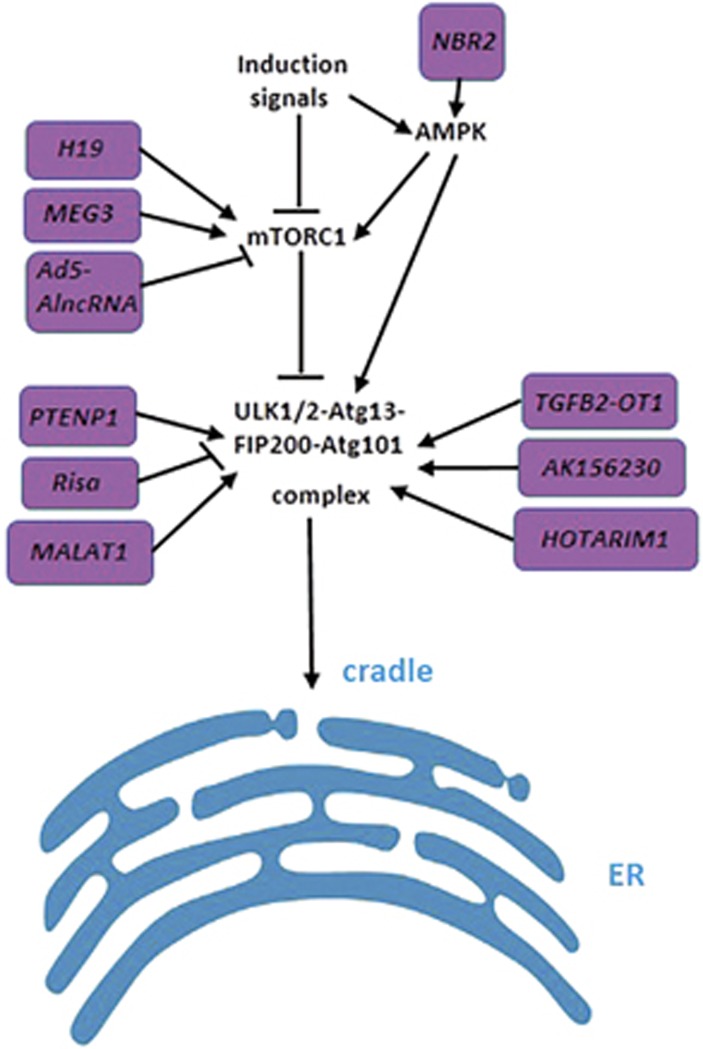
Brief summarization of the initiation step of mammalian core autophagy machinery and its regulation by lncRNAs. Upon energy limitation, autophagy can be initiated by AMPK and mTORC1. Then, ULK1 complex composed of ULK1, ATG13, FIP200 and ATG101 is activated. lncRNA *NBR2* directly modulates AMPK and mTORC1 are positively regulated by *MEG3* and *H19* and negatively regulated by *Ad5-AlncRNA*. In addition, lncRNA *PTENP1*, *Risa*, *MALAT1*, *TGFB2-OT1*, *AK156230* and *HOTARIM1* collectively regulate ULK1 complex

As summarized in Figure 2 and Table 1, the lncRNA *NBR2* promotes autophagy initiation by directly activating AMPK.^36^ The lncRNAs *Ad5-AlncRNA*^45^ *and PTENP1*^58^ provoke autophagy initiation by repressing the PI3K/AKT/mTOR pathway, whereas *MEG3*^47^ and *H19*^33^ has the opposite effect. The lncRNAs *HOTAIRM1*,^53^ *PTENP1*^58^ and *MALAT1*^71^ enhance autophagy initiation by increasing ULK expression. Meanwhile, the lncRNA *Risa* negatively regulates autophagy initiation by inhibiting ULK1 activation.^72^ In addition, the lncRNAs *H19*,^33^ *MEG3*,^50^ *AK156230*,^64^ *PTENP1*^58^ and *MALAT1*^71^ can also influence other *ATG* genes and autophagy adaptor proteins involved in later steps of autophagy regulation.

### LncRNAs related to phagophore nucleation

Once activated and translocated to PAS/omegasome, the ATG1/ULK1 complex can activate the class III PI3K complex, which mainly comprises Vps34, Vps15, vps30/Beclin 1 (yeast/mammal) and ATG14/Barkor (yeast/mammal), to generate phosphatidylinositol 3-phosphate (PI3P).^73, 74, 75^ PI3P recruits double FYVE-containing protein 1 (DFCP1) to promote the formation of the omegasome.^76^ Meanwhile, other essential regulators such as ATG9, ATG18 (WIPI1/2/3/4) and VMP1 are present on the autophagic membrane.^30, 77^ During the process of class III PI3K complex formation and function, BCL-2 and Rubicon act as two negative regulators.^78, 79^

Two studies have demonstrated that the inhibition of *LincRNA, regulator of reprogramming* (*Linc-ROR)* can elicit autophagy by upregulating Beclin 1 expression, and revise gemcitabine and tamoxifen resistance in breast cancer respectively.^80, 81^ However, further experiments need to reveal how *Linc-ROR* regulates Beclin 1 expression and whether knockdown of *Linc-ROR* could be feasible in clinical practice.

LncRNA *loc146880* reveals to be intricately related to autophagy and the biogenesis of lung cancer.^82, 83^ High expression of reactive oxygen species (ROS) induced by PM2.5 (a class of particulate matters, <2.5*μ*m in diameter) exposure enhances lncRNA *loc146880* expression, which results in autophagy activation and subsequently contributes to lung cell migration and invasion. *Beclin 1* mRNA is upregulated along with the increase of *loc146880* expression, but the underlying mechanism is far from fully explained.^83^

LncRNA *AC023115.3* is upregulated by cisplatin and promotes cisplatin-induced apoptosis by impeding autophagy in glioma. Further mechanistic studies reveal that *AC023115.3* can elevate GSK3*β* expression by sponging miR-26a.^84^ GSK3*β* promotes the degradation of Mcl1, a member of BCL-2 family that sequesters Beclin 1 from the class III PI3K complex.^85^ Further studies are needed to address whether Beclin 1 will activate the class III PI3K complex to a greater extent upon overexpression of *AC023115.3* as hypothesized. Regardless, the study reveals that the *AC023115.3*/miR-26a/GSK3*β* signaling cascade has a significant role in promoting chemosensitivity in gliomas and serves as an exciting indicator to exploit the association between *AC023115.3* and Beclin 1.

**Figure 3:**
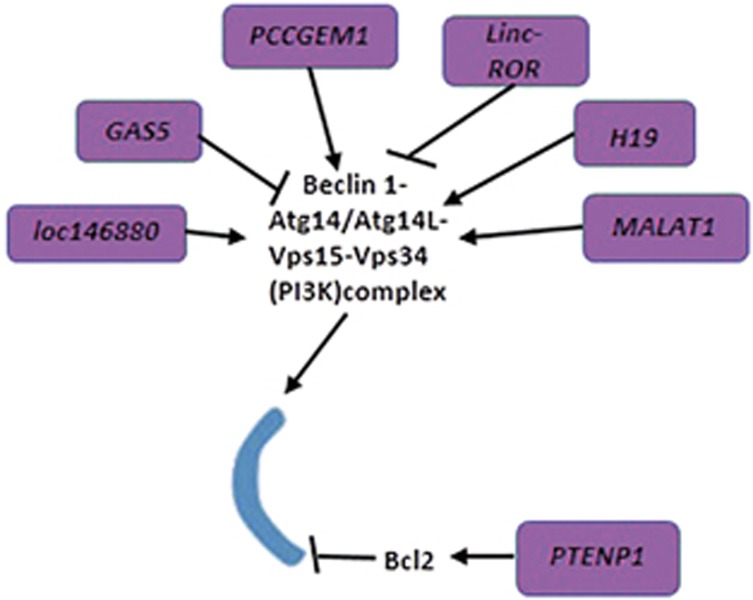
Brief summarization of the vesicle nucleation step of mammalian core autophagy machinery and its regulation by lncRNAs. Vesicle nucleation is predominantly modulated by class III PI3K complex, which comprises Vps34, Vps15, Beclin 1 and ATG14/ATG14L. During the process of PI3K complex formation and function, BCL-2 and Rubicon act as two negative regulators. lncRNA *GAS5*, *PCGEM1* and *Linc-ROR* regulate PI3K complex, among which, Beclin 1 itself is positively regulated by lncRNA *PCGEM1*, *MALAT1*, *H19* and *loc146880*, whereas negatively regulated by *GAS5*, *Linc-ROR* and *H19*. Moreover, lncRNA *PTENP1* can repress autophagy via increasing BCL-2 expression

Collectively, both *Linc-ROR* and *loc146880* can impact vesicle nucleation by modulating Beclin 1 gene or protein expression^81, 83^ (Figure 3; Table 1). In addition, the association between AC023115.3 and Beclin 1 still needs further investigation.

### lncRNAs related to autophagosome elongation/closure

The two unique ubiquitin-like conjugation systems have crucial roles in the elongation and closure of the isolated membrane. Driven by ATG7 (E1-like enzyme) and ATG10 (E2-like enzyme), ATG12 conjugates to ATG5 and then interacts with ATG16 (mammal ortholog is ATG16L) to form the ATG12-ATG5-ATG16 complex.^86, 87, 88^ Subsequently, the ATG12-ATG5-ATG16 complex, ATG7 and ATG3 (E2-like enzyme) jointly transform ATG8 (LC3 in mammals) from its cytosolic soluble isoform (LC3-I) to its membrane-anchored isoform (LC3-II).^23^ In addition, adaptor proteins such as ATG19 (an adaptor for the Cvt pathway) and ATG32 in yeast, as well as the neighbor of BRCA1 gene 1 (NBR1), Nix (also called Bnip3L) and p62 in mammals, selectively mediate the degradation of proteins or organelles by recruiting them to autophagosomes via binding to LC3-II.^31^

A study reveals that the lncRNA *TGFB2* overlapping transcript 1 (*TGFB2-OT1,* also known as *FLJ11812*) can be upregulated by vascular endothelial cell (VEC) inflammation triggers and function as the sponge for miR-3960, miR-4488 and miR-4459, thereby increasing the expression levels of their targets, such as *ATG13*, ceramide synthase 1 (CERS1) and La ribonucleoprotein domain family, member 1 (LARP1). In addition, overexpression of *TGFB2-OT1* can also increase ATG3, ATG7 and p62 expression, probably through upregulating LARP1 by sponging miR-4459, an RNA-binding protein related to transcript stability and translation of mRNAs.^89, 90^ Moreover, when *TGFB2-OT1* prevents miR-3960-mediated repression of CERS1, production of C18-ceramide is increased to induce mitophagy (a class of selective autophagy targeting dysfunctional mitochondria) by directly interacting with LC3-II-containing autophagolysosomes upon Drp1-dependent mitochondrial fission.^89, 91^ In summary, ectopic expression of *TGFB2-OT1* induced by VEC inflammation triggers activates autophagy via increasing ATG13, ATG3, ATG7 and p62 expression, and a small molecular inhibitor 3BDO significantly decreases *TGFB2-OT1* level and inhibits the subsequent autophagic and inflammatory reaction.^89^ Given that autophagy and inflammation have an intricate correlation, intervening in *TGFB2-OT1* could be a possible treatment strategy for infectious and autoimmune diseases.^92^

The lncRNA *growth arrest-specific 5* (*GAS5*) has been reported to inhibit autophagy and enhance cisplatin sensitivity in NSCLC cells.^93^ In contrast to its deletion in several species of tumors, GAS5 is upregulated in osteoarthritis (OA), repressing autophagy and stimulating the apoptosis of OA chondrocytes, which is a key determinant responsible for cartilage degradation and thus OA pathogenesis.^94^ High expression of *GAS5* in OA represses autophagy possibly through downregulating Beclin 1, ATG3, ATG5, ATG7, ATG12 and LC3B expression.^94^ It seems that *GAS5*-repressed autophagy is beneficial for enhancing drug sensitivity but results in the occurrence of OA. Therefore, different modes of interference with GAS5 may be essential for distinct therapeutic purposes.

The lncRNA *HNF1A-AS1* can sequester miR-30b from binding to its target *ATG5* and thereby provoke autophagy in HCC.^95^ In addition, *Beclin 1* and *ATG12* have also been defined as targets of miR-30b in a previous study, indicating that, aside from *ATG5*, *HNF1A-AS1* might also upregulate Beclin 1 and ATG12 expression to promote vesicle nucleation and autophagosome elongation/closure.^95, 96^ Considering that autophagy was previously confirmed to promote HCC, it is likely that *HNF1A-AS1* facilitates HCC biogenesis by promoting autophagy.^97, 98, 99^

Exogenous overexpression of the lncRNA, *prostate cancer gene expression marker 1* (*PCGEM1*), can increase the mRNA levels of *Beclin 1*, *ATG3*, *ATG5* and *ATG12*, indicating that PCGEM1 may be involved in the induction of autophagy, and promote the proliferation of human synoviocytes.^100^ PCGEM1 expression is associated positively with mortality rate in African-American patients with prostate cancer, probably because of PCGEM1-induced autophagy.^101^ However, further analysis is required to confirm whether and how *PCGEM1* affects autophagy.

Mounting evidences reveal that LncRNAs harbor much more tissue and developmental stage specificity than coding transcripts.^102^ In line with this discovery, lncRNA highly upregulated in liver cancer (*HULC*) has been found to predominantly express in primary HCC and metastatic hepatic carcinoma.^103^ One study elucidates clearly how the '*HULC*/USP22/Sirt1/protective autophagy' pathway attenuates the chemosensitivity of HCC.^104^ In detail, *HULC* can upregulate ubiquitin-specific peptidase (USP22) expression level via repressing miR-6825-5p, miR-6845-5p and miR-6886-3p, and ectopic expression of USP22 can stabilize Sirt1 protein, which promotes protective autophagy by deacetylating Atg5 and Atg7.^104^ Mazy as it seems, this *HULC*-regulated autophagy pathway makes a promising target to address chemoresistance in HCC. Another work indicates that *HULC* overexpression increases proliferation and invasion of gastric cancer (GC) cells probably through arousing autophagy activation.^105^ Clearly, these findings put forward a new topic worthy of studying that how *HULC* provokes autophagy in GC and whether *HULC* can be identified as a biomarker both in HCC and GC.

In anoxia/reoxygenation (A/R)-induced autophagy of cardiomyocytes, the lncRNA autophagy promoting factor (*APF*) is upregulated to protect *ATG7* from being downregulated by miR-188-3p, and thereby promotes autophagic death of cardiomyocytes.^106^ Intriguingly, despite the poor conservation of full-length *APF* across species, the binding site for miR-188-3p is highly conserved, highlighting the significance of the *APF*/miR-188-3p/*ATG7* regulatory axis in autophagy activation.^106^

The lncRNA *HOTAIR* is well characterized for recruiting the epigenetic modification factors, such as polycomb repressive complex 2 (PRC2), to regulate gene expression, thereby promoting tumor cell proliferation and migration.^11, 107^ Analogously, *HOTAIR* is upregulated in chondrosarcoma and induces DNA methylation of miR-454-3p promoter regions by recruiting EZH2 and DNA methyltransferase 1 (DNMT1), which markedly silences miR-454-3p expression. *ATG12* and STAT3 are targets of miR-454-3p, providing one molecular dissection of *HOTAIR* deficiency-induced autophagy repression and apoptosis.^108^ Another study demonstrates that *HOTAIR* is upregulated in HCC to promote HCC cell proliferation, probably by enhancing ATG3 and ATG7 expression to expedite autophagy flux.^109^ As *HOTAIR* can interact with numerous miRNAs, such as miR-34a, miR-331-3P, miR-130a and miR-454-3p, we should recall that *HOTAIR* regulates autophagy in two ways. First, *HOTAIR* may serve as a scaffold to recruit epigenetic modification enzymes to inhibit miRNA transcription; second, *HOTAIR* can act as a sponge to sequester miRNAs from their targets. In both cases, *HOTAIR* will upregulate specific genes targeted by corresponding miRNAs, possibly including some *ATG* genes. For instance, miR-130a can repress the transcription of *ATG5* and *ATG16L,* and *Atg12* is a target of miR-454-3p.^12, 108, 110, 111, 112, 113, 114^

**Figure 4:**
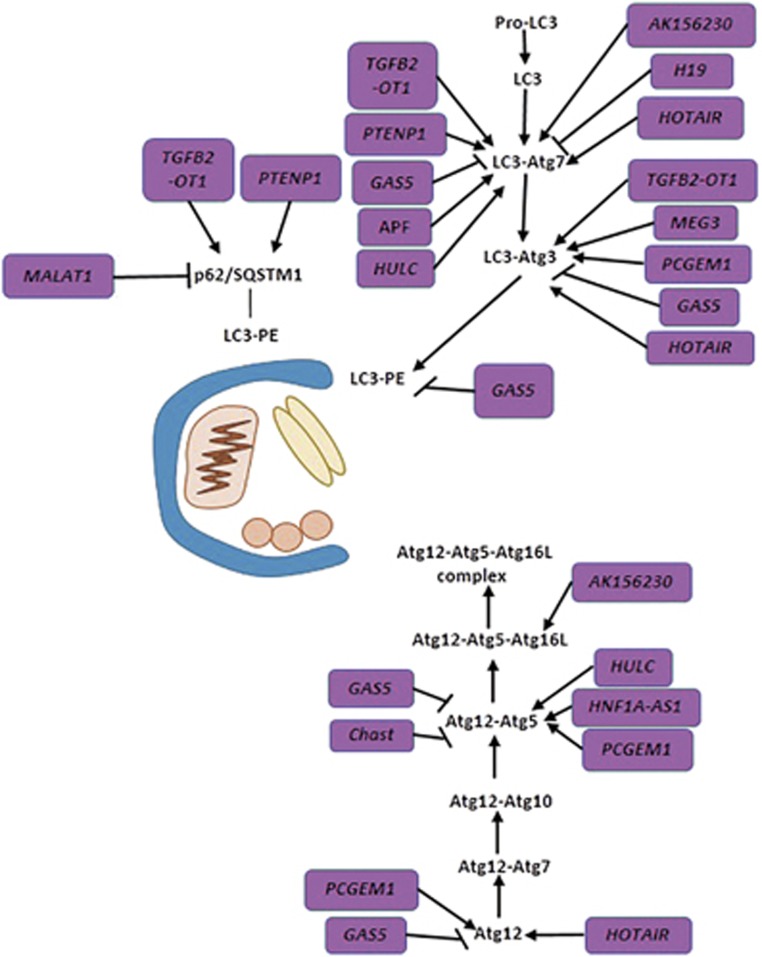
Brief summarization of the autophagosome elongation/closure step and its regulation by lncRNAs. Autophagosome elongation/closure is controlled by two ubiquitin-like conjugation systems and scaffold proteins, such as p62. ATG12-ATG5-ATG16L complex, ATG7 and ATG3 jointly transform LC3-I to LC3-II. Meanwhile, p62 selectively mediates the degradation of proteins or organelles by recruiting them to autophagosome via its binding to LC3. Numerous lncRNAs are involved in this process, containing *TGFB2-OT1*, *PTENP1*, *GAS5*, *APF*, *AK156230*, *HOTAIR, HULC* and *H19* (ATG7), *MEG3*,*TGFB2-OT1*, *PCGEM1*, *GAS5* and *HOTAIR* (ATG3), *HNF1A-AS1*, *PCGEM1*, *GAS5*, *HULC* and *Chast* (ATG5), *PCGEM1, HOTAIR* and *GAS* (ATG12),*AK156230* (ATG16L), *PTENP1, TGFB2-OT1* and *MALAT1* (p62)

Taken together, as shown in Figure 4 and Table 1, the lncRNAs *TGFB2-OT1*,^89, 90^ *GAS5*,^94^ *HNF1A-AS1*,^95, 96^ *PCGEM1*,^100^ *HULC,*^104^ *APF*^106^ and *HOTAIR,*^108, 109^ promote autophagosome elongation/closure by elevating the expression of *ATG* genes involved in the ubiquitin-like conjugation systems. Meanwhile, the lncRNA *GAS5*^94^ may repress and the lncRNA *PCGEM1*^100^ may activate autophagy nucleation by interacting with Beclin 1. The lncRNA *TGFB2-OT1* activates autophagy initiation by increasing ATG13 expression.^89^

### LncRNAs related to autolysosome fusion

The final step of autophagy flux is the fusion of the autophagosome and the lysosome to form an autolysosome, where the autophagic cargo is degraded.^31^ The core molecules in this stage include the Rab-SNARE system and the lysosome membrane proteins LAMP1 and LAMP2.^115, 116, 117, 118^ In addition, adaptor proteins are necessary to link endocytic and autophagic pathways to the lysosome. Pleckstrin homology domain-containing protein family M member 1 (Plekhm1) is one such adaptor protein, which contains an LC3-interacting region and directly interacts with the homotypic fusion and protein sorting complex to mediate the fusion of endosomes and autophagosomes with lysosomes.^119^

**Figure 5:**
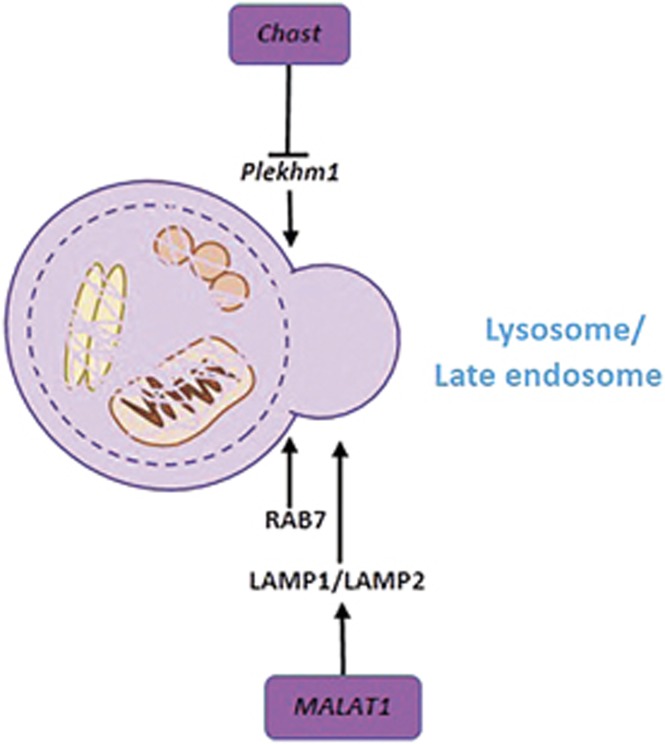
Brief summarization of the autolysosome fusion and its regulation by lncRNAs. Fusion is the final step that autophagosome fuses with endosome and lysosome to form autolysosome, where the autophagic cargo is degraded. The lncRNA chast and MALAT1 have been found to regulate the step

The lncRNA cardiac hypertrophy-associated transcript (*Chast*) can suppress autophagy via downregulating Plekhm1 and possibly ATG5 expression to induce cardiomyocyte hypertrophy (Figure 5).^120^ This study sheds light on the mechanism of Chast/Plekhm1 interaction during autolysosome fusion and implies that Chast is a possible target for the prevention of cardiac remodeling.

## Additional lncRNA Regulators of Autophagy

Some lncRNAs, including lncRNA *7SL*,^121^ *BANCR,*^122^ *PCA3,*^123^ *LINC01116*^124^ and *CTA*,^125^ also have been found to be related to autophagy, although the evidence of their correlation with *ATG* genes is lacking. *BANCR*^122^ can provoke autophagy flux to facilitate tumor proliferation, whereas *7SL*,^121^ *PCA3*,^123^ *LINC01116*^124^ and *CTA*^125^ have the opposite effect.

## LncRNAs Regulated by Autophagy

As an increasing number of lncRNAs have been identified to regulate autophagy, it would be interesting to know whether autophagy could also affect the expression of lncRNAs. Autophagy can degrade several types of RNAs and associated ribonucleoprotein complexes.^126^ *Plasmacytoma variant translocation 1* (*PVT1*) is the sole lncRNA reported to be regulated by autophagy so far. *PVT1* is upregulated in diabetes, and autophagy repression decreases its transcriptional level.^127^ The elevation of *PVT1* mediated by autophagy functions crucially in the development and progression of diabetic nephropathy.^128, 129^ Clearly, PVT1 is probably not degraded by autophagy, as it is downregulated when autophagy is repressed.^127^ Thus, extensive further investigations are needed to demonstrate what determinants participate in this process.

## Conclusions

Given the limitations of the research that has been conducted to date, we have gained limited knowledge of the underlying mechanisms of regulation between identified lncRNAs and autophagy. The majority of lncRNAs typically function as sponges to sequester autophagy-related miRNAs from their targets.^130, 131^ Certainly, lncRNAs have more complicated functions in autophagy regulation that are waiting to be elucidated, including but not limited to chromatin and histone remodeling, transcriptional regulation and protein–protein interactions.^132^ Current studies also indicate that we may need to classify lncRNAs according to their roles in distinct types of autophagy, such as mitophagy, to probe their function more specifically.^47, 89^ From the growing knowledge based on lncRNAs and autophagy, we have formed the impression that most lncRNAs involved in autophagy have parts in tumorigenesis and that this function can be interpreted through their association with autophagy. In consideration of the intimate linkage between lncRNAs and autophagy, it would be possible to develop lncRNA-based approaches to monitor or interfere with autophagy flux. As lncRNA expression is prevalent along the developmental and spatial axis, and several autophagy-associated lncRNAs described in this review virtually exhibit tissue specificity, such as *HULC* and *PCA3*, lncRNAs may serve as biomarkers of specific diseases, and pertinent therapeutic measures may be developed.^103, 133^ Finally, both lncRNAs and autophagy are involved in a vast range of diseases including cancer; therefore, a joint intervention targeting lncRNAs and autophagy may be a promising therapeutic method.

## Figures and Tables

**Table 1:** lncRNAs involved in autophagy regulation

**Autophagy stages**	**Relevant autophagy-related genes or proteins**	**lncRNA**
Initiation	AMPK	*NBR2*^36, 37^
	mTORC1	*Ad5-AlncRNA*^45^*MEG3*^47^*H19*^33^
	ATG13	*TGFB2-OT1/FLJ11812*^89, 90^
	ULK1/2	*PTENP1*^58, 59, 60^ *Risa*^72^ MALAT1^71^ *HOTAIRM1*^53, 134, 135^ *AK156230*^64^
Vesicle nucleation	Beclin 1	*PCGEM1*^100^*H19*^33, 136^*GAS5*^94^ *MALAT1*^69^ *loc146880*^83^*Linc-ROR*^81^
	Bcl-2	*PTENP1*^58, 59, 60^
Autophagasome elongation/closure	ATG7	*TGFB2-OT1/FLJ11812*^89, 90^ *AK156230*^64^ *PTENP1*^58, 59, 60^ *GAS5*^94^*APF*^106^*HOTAIR*^109^ *H19*^33^*HULC*^104^
	ATG5	*PCGEM1*^100^*HNF1A-AS1*^95, 137^*Chast*^120^*GAS5*^94^ *HULC*^104^
	ATG3	*MEG3*^50^ *TGFB2-OT1/FLJ11812*^89, 90^ *PCGEM1*^100^ *GAS5*^94^ *HOTAIR*^109^
	ATG12 ATG16L	*PCGEM1*^100^*HNF1A-AS1*^27, 95^*GAS5*^94^*HOTAIR*^108^ *AK156230*^64^
	p62	*PTENP1*^58, 59, 60^ *MALAT1*^66^
Fusion	Plekhm1 LAMP2	*Chast*^120^ *MALAT1*^66^
